# Alopecia areata severity and patient-reported intimacy: A cross-sectional survey study

**DOI:** 10.1016/j.jdin.2025.04.013

**Published:** 2025-06-11

**Authors:** Aaron Bao, Joy Jin, Cynthia Smith, Marwa Hakimi, Kala M. Mehta, Tina Bhutani, Jennifer Fu

**Affiliations:** a Department of Dermatology, The Johns Hopkins University School of Medicine, Baltimore, Maryland; b Department of Dermatology, University of California San Francisco, San Francisco, California; c Solano Dermatology Associates, Fairfield, California

**Keywords:** alopecia, alopecia areata, hair, other, psychology/psychiatry, quality of life, survey study

*To the Editor:* Alopecia areata (AA) affects approximately 2% of the global population, with significant psychosocial burden.^1^ While AA's impact on quality of life is well-documented, its effects on intimacy remain understudied.^2^ To address this gap, we conducted a cross-sectional survey study to investigate how disease severity and sociodemographic factors influence intimacy among AA patients (Supplementary Method 1, available via Mendeley at https://data.mendeley.com/datasets/xwtmcnxydr/1).

Web-based survey administration was approved by the National Alopecia Areata Foundation (NAAF). Patients with AA were recruited through the NAAF website and email newsletter between September 2022 and March 2023. The primary outcome was the dermatology intimacy scale (DIS),^3^ an 18-item questionnaire originally validated in psoriasis, which measures skin conditions' impact on intimacy (range 0-72, higher scores indicating greater negative impact) (Supplementary Text 1, available via Mendeley at https://data.mendeley.com/datasets/xwtmcnxydr/1). Participants estimated scalp involvement as none-to-limited (0% to 20%), moderate (21% to 49%), severe (50% to 94%), or very severe (95% to 100%). Associations between hair loss categories and DIS scores were assessed through Kruskal-Wallis tests. Univariable and adjusted multivariable linear regression was performed to analyze the independent effects of sociodemographic and clinical characteristics on DIS scores.

Of 907 survey invitations, 706 (78%) completed responses were included in the study, excluding patients <18 years, without a physician-provided AA diagnosis, or with incomplete outcome data. Most participants were female (83.7%), White (75.5%), and married (54.5%), with a median (IQR) age of 53 (23) years (Supplementary Table I, available via Mendeley at https://data.mendeley.com/datasets/xwtmcnxydr/1). A total of 47.2% reported very severe scalp involvement, with 64.5% indicating their current episode was their worst. A total of 25.7% and 19.8% reported complete or partial loss of eyebrow/eyelash and groin hair, respectively. The median (IQR) DIS score was 31 (38), and the median (IQR) disease duration was 14 (22) years (Table I). Relative to those with none-to-limited (0% to 20%) loss, participants with greater severity had worse DIS scores (*P* < .05 for all) (Fig 1).

**Table I:** Clinical characteristics and outcome dermatology intimacy scale scores of study participants

Clinical characteristics (*N* = 706)	
Age of AA diagnosis, median (IQR), y	31 (31)
Duration of AA diagnosis, median (IQR), y	14 (22)
Current extent of missing scalp hair, *n* (%)	
Very severe (95% to 100%)	331 (47)
Severe area (50% to 94%)	103 (15)
Moderate area (21% to 49%)	87 (12)
None-to-limited area (0% to 20%)	176 (25)
Unsure or missing	9 (1)
Current presence of alopecia universalis∗, *n* (%)	
Yes	292 (42)
No	399 (57)
Unsure or missing	15 (2)
Current loss of eyebrow and/or eyelash hairs, *n* (%)	
Complete loss	47 (7)
Partial loss	135 (19)
No loss	214 (31)
Unsure or missing	310 (44)
Current loss of groin hairs, *n* (%)	
Complete loss	16 (2)
Partial loss	124 (18)
No loss	250 (36)
Unsure or missing	316 (45)
Length of current episode of hair loss, *n* (%)	
≥12 mo	532 (76)
<12 mo	140 (20)
Unsure or missing	34 (4)
Is it currently your worst, most severe episode?, *n* (%)	
Yes	456 (65)
No	222 (31)
Unsure or missing	28 (4)

Outcomes (*N* = 706)	
Total DIS score, median (IQR)	31 (38)
Total DIS score, mean (SD)	32 (22)

*AA*, Alopecia areata; *DIS*, dermatology intimacy scale; *IQR*, interquartile range.

∗ Defined as complete loss of hair on scalp and body.

**Fig 1:**
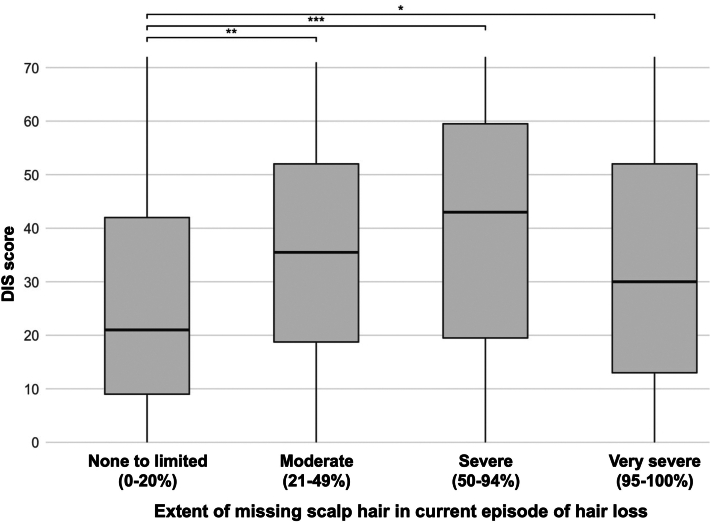
Relationship between extent of scalp hair loss and dermatologic intimacy scale (DIS). Box plots display median (*black line*), interquartile range (*boxes*), and individual data points, with *asterisks* indicating statistically significant differences between groups (∗, *P* < .05; ∗∗, *P* < .01; ∗∗∗, *P* < .001) based on Kruskal-Wallis testing.

Severe (50% to 94%) scalp involvement showed the strongest association with worse DIS scores (β = 12.3; *P* < .001), followed by very severe (95% to 100%) (β = 6.9; *P* = .002) and moderate (21% to 49%) involvement (β = 6.2; *P* = .04). Single relationship status (β = 12.7; *P* < .001), female sex (β = 8.7; *P* < .001), Black or African American race (β = 7.7; *P* = .003), shorter disease duration (β = −0.14; *P* = .01), and younger age (β = −0.13; *P* = .02) were independently associated with worse scores. Alopecia universalis (β = 4.4; *P* = .02) and currently experiencing one's worst episode (β = 4.9; *P* = .007) also correlated with worse DIS scores (Supplementary Table II, available via Mendeley at https://data.mendeley.com/datasets/xwtmcnxydr/1).

Our findings suggest a complex relationship between disease involvement and intimacy. Notably, moderate hair loss was associated with impaired intimacy scores comparable to very severe loss, supporting expert consensus that moderate scalp involvement with psychosocial impairment should be classified as having severe disease.^4^^,^^5^ This may reflect unique challenges faced in partial, visible hair loss – unpredictable appearance, difficult concealment, and heightened self-consciousness.

Study limitations include selection bias from NAAF recruitment yielding a predominantly White sample and likely excluding individuals disengaged from support organizations. Without controls, we cannot definitively conclude whether observed effects are AA-specific or represent general concerns associated with visible dermatological conditions. Given AA’s differing disease course and coping strategies, use of a psoriasis-validated intimacy scale may also affect DIS score interpretation. Lastly, unmeasured confounders like psychiatric comorbidities or cultural perceptions also limit our findings. Nevertheless, our results support routine screening for intimacy concerns, particularly among single individuals, women, racial minorities, and newly diagnosed or younger patients. Early psychosocial support may benefit patients with progressive disease. Future studies should validate these findings in larger, more representative cohorts while examining the temporal relationship between disease progression and intimacy impacts.

## Conflicts of interest

None disclosed.

## References

[1] Villasante Fricke A.C., Miteva M. (2015). Epidemiology and burden of alopecia areata: a systematic review. Clin Cosmet Investig Dermatol.

[2] Mesinkovska N., King B., Mirmirani P., Ko J., Cassella J. (2020). Burden of illness in alopecia areata: a cross-sectional online survey study. J Investig Dermatol Symp Proc.

[3] Malakouti M., Brown G.E., Leon A. (2017). The dermatologic intimacy scale: quantitatively measuring the impact of skin disease on intimacy. J Dermatolog Treat.

[4] King B.A., Mesinkovska N.A., Craiglow B. (2022). Development of the alopecia areata scale for clinical use: results of an academic-industry collaborative effort. J Am Acad Dermatol.

[5] Nohria A., Zhang Y.C., Desai D. (2024). Real-world experience quantifying access to JAK inhibitor care for alopecia areata patients: a patient-centered survey study. Int J Womens Dermatol.

